# Enhancing mitochondrial one-carbon metabolism is neuroprotective in Alzheimer’s disease models

**DOI:** 10.1038/s41419-024-07179-3

**Published:** 2024-11-24

**Authors:** Yizhou Yu, Civia Z. Chen, Ivana Celardo, Bryan Wei Zhi Tan, James D. Hurcomb, Nuno Santos Leal, Rebeka Popovic, Samantha H. Y. Loh, L. Miguel Martins

**Affiliations:** 1 MRC Toxicology Unit, University of Cambridge, Gleeson Building, Tennis Court Road, Cambridge, CB2 1QR UK; 2 Healthspan Biotics Ltd, Milner Therapeutics Institute, Cambridge Biomedical Campus, Cambridge, UK; 3 Wellcome-Medical Research Council Cambridge Stem Cell Institute, Cambridge Biomedical Campus, University of Cambridge, Cambridge, UK

**Keywords:** Alzheimer's disease, Energy metabolism

## Abstract

Alzheimer’s disease (AD) is the most common form of age-related dementia. In AD, the death of neurons in the central nervous system is associated with the accumulation of toxic amyloid β peptide (Aβ) and mitochondrial dysfunction. Mitochondria are signal transducers of metabolic and biochemical information, and their impairment can compromise cellular function. Mitochondria compartmentalise several pathways, including folate-dependent one-carbon (1C) metabolism and electron transport by respiratory complexes. Mitochondrial 1C metabolism is linked to electron transport through complex I of the respiratory chain. Here, we analysed the proteomic changes in a fly model of AD by overexpressing a toxic form of Aβ (Aβ-Arc). We found that expressing Aβ-Arc caused alterations in components of both complex I and mitochondrial 1C metabolism. Genetically enhancing mitochondrial 1C metabolism through *Nmdmc* improved mitochondrial function and was neuroprotective in fly models of AD. We also found that exogenous supplementation with the 1C donor folinic acid improved mitochondrial health in both mammalian cells and fly models of AD. We found that genetic variations in *MTHFD2L*, the human orthologue of *Nmdmc*, were linked to AD risk. Additionally, Mendelian randomisation showed that increased folate intake decreased the risk of developing AD. Overall, our data suggest enhancement of folate-dependent 1C metabolism as a viable strategy to delay the progression and attenuate the severity of AD.

## Introduction

Alzheimer’s disease (AD), an age-associated neurodegenerative disease, is projected to affect over 150 million people globally by 2050 [1]. Some drugs are available to slow cognitive decline in patients (reviewed in [2]), but there is no cure for AD. Therefore, new strategies are needed to mitigate AD progression and severity. Familial forms of AD are associated with an increased aggregation of Aβ in the brain [3–6]. After over 100 failed clinical trials [7, 8], a recent clinical trial using lecanemab [9], an antibody developed to clear Aβ, showed promising results. However, other strategies that use antibodies to clear this toxic protein from AD brains have failed [10]. Due to the uncertainty around the efficacy and safety of therapeutic strategies focused on clearing Aβ, additional approaches are needed to treat AD, including multitarget therapies. Mitochondria are central players in energy generation and overall cellular physiology, and mitochondrial dysfunction is widely reported to occur in models of AD. However, this is thought to be secondary to the other well-recognised pathogenic mechanisms in AD (reviewed in [11]).

*Drosophila melanogaster* is a powerful animal model for research on the mechanisms of neurodegeneration and identification of ways to delay or prevent it [12]. *Drosophila* models of AD are created by expressing toxic human Aβ in fly neurons [13]. The neuronal expression of Aβ-Arc, consisting of the disease-associated Aβ(1–42) peptide with the Arctic mutation (Glu22Gly), causes neurodegeneration and premature neuronal cell death, recapitulating pathological features of AD in humans (reviewed in ref. [14]). Neuronal expression of Aβ-Arc also leads to mitochondrial toxicity in *Drosophila* [15], providing an opportunity to study the mechanisms by which mitochondria contribute to AD pathology.

Mitochondria compartmentalise several biochemical pathways, including the oxidative phosphorylation (OXPHOS) system and a branch of folate-dependent one-carbon (1C) metabolism. 1C metabolism requires one of three cofactors, biotin, tetrahydrofolate (THF) or S-adenosylmethionine (SAM), to transfer 1C groups to various biosynthetic pathways. One of the critical components of 1C metabolism is the folate cycle. Folate-dependent 1C metabolism uses THF as a carbon donor to fuel mitochondrial enzymes with electrons and produce purines. In mitochondria, a multienzyme complex containing the NAD-dependent methylenetetrahydrofolate dehydrogenase-methenyltetrahydrofolate cyclohydrolase (Nmdmc), a bifunctional enzyme also known as mitochondrial methylenetetrahydrofolate dehydrogenase (MTHFD2L), catalyses the formation of 5,10-methenyl-THF and 10-formyl-THF. This two-step catalytic reaction is linked to the transport of electrons by complex I (NADH:ubiquinone oxidoreductase), a component of the OXPHOS system. The NADPH produced via oxidation of 10-formyl-THF to carbon dioxide (CO_2_) is proposed to transfer electrons to NAD^+^, which is then used by complex I (reviewed in ref. [16]). Nmdmc can reduce both NAD^+^ and NADP^+^ as substrates during the oxidation of 5,10-methenyltetrahydrofolate [17]. 1C metabolism has thus been linked to neurodegenerative diseases including AD (reviewed in ref. [18]), but the specific mechanisms remain uncertain. Specifically, the connection between the mitochondrial compartment of 1C metabolism and AD is unknown.

Here, we explored the global protein changes due to the expression of Aβ-Arc in flies and identified alterations in components of 1C metabolism and complex I. We show that expression of Aβ-Arc is linked to reduced complex I activity and that complex I activity can be rescued by increasing mitochondrial folate-dependent 1C metabolism in models of AD. We also describe links between this mitochondrial 1C pathway and the risk of developing AD, using human data. Our results suggest that enhancing folate-dependent 1C metabolism is a potential strategy for delaying the progression and reducing the severity of AD.

## Results

### Adult flies expressing toxic Aβ show increased levels of mitochondrial respiratory complex I and components of folate metabolism

**Fig. 1:**
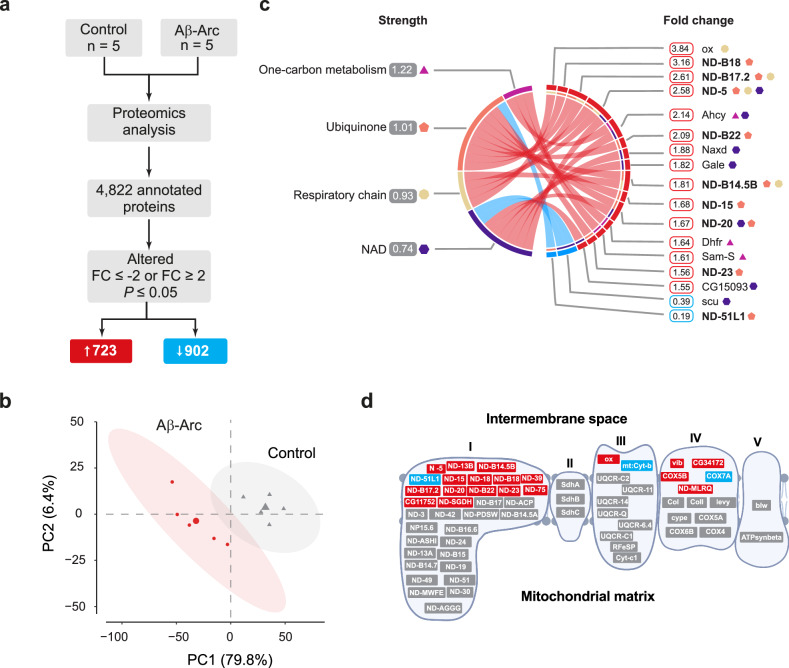
Expression of a toxic form of Aβ increases components of 1C metabolism and mitochondrial respiration. **a** Workflow employed for the identification of protein changes in adult flies expressing toxic Aβ-Arc. For each genotype, proteins from 5 samples of 30 male flies aged for 10 days after eclosion were compared. Significance was determined using a linear mixed model moderated by empirical Bayes methods. Multiple comparisons were adjusted using the Benjamin‒Hochberg method. **b** PCA of the proteins that were altered upon expression of toxic Aβ-Arc in adult flies. **c** Pathway analysis of the top proteins (299 proteins) selected by PC1 in the unsupervised PCA. The analysis was performed based on UniProt functional enrichment (see also Materials and Methods). Significant pathways are on the left side, and only proteins linked to the enriched pathways are shown (17 proteins). The symbols beside the protein names indicate the pathway they belong to. Protein subunits of mitochondrial respiratory complex I are highlighted in bold. All protein levels were significantly changed, with the proteins with increased levels in red and those with decreased levels in blue, respectively. **d** Alterations in protein composition of mitochondrial respiratory complex, mapped at the individual subunit level of the respiratory complexes (I, II, III or IV). Red and blue indicate subunits with protein levels that are, respectively, increased and decreased. Subunits labelled in grey represent protein levels that were not significantly altered. Genotypes: *w; +; daGal4/+* (Control)*, w; UAS Aβ42Arc/+; daGal4/+* (Aβ-Arc).

Expression of toxic Aβ-Arc in adult flies causes metabolic changes, including reductions in the levels of NAD^+^, a coenzyme central to metabolism that is required for electron transfer in redox reactions [15]. To define the protein changes linked to the altered NAD^+^ metabolism in this fly model of AD, we first measured the global protein levels in the flies using quantitative proteomics. This analysis (Fig. 1a) showed that expression of toxic Aβ-Arc led to alterations in 1578 (~33%) out of 4822 proteins that were detected (Fig. 1a and Supplemental Table 1), including a network of mitochondrial proteins linked to ubiquinone (FDR < 0.0001, https://m1gus.github.io/AD-FA/). Principal component analysis (PCA) of the global proteomic alterations showed that the first principal component (PC1) accounted for 79.8% of the variance between Aβ-Arc-expressing flies and controls (Fig. 1b). Since PC1 accounted for most of the proteomic alterations caused by the expression of toxic Aβ-Arc, we conducted functional enrichment analysis (see Methods and Fig. 1c) of the top contributors in PC1. This showed that Aβ-Arc expression led to upregulation of several protein subunits of mitochondrial NADH:ubiquinone oxidoreductase (complex I, Fig. 1d), the entry point for electrons into the respiratory chain, as well as several components of NAD^+^ and 1C metabolism (Fig. 1c). We found increases in the levels of dihydrofolate reductase (Dhfr), an enzyme that uses NADPH as an electron donor to reduce dihydrofolic acid to THF, which is then used in the folate pathway of 1C metabolism. We also observed increases in the levels of other components of 1C metabolism: adenosylhomocysteinase (Ahcy13), which uses NAD^+^ as a coenzyme, and S-adenosyl-methionine synthase, which is involved in the generation of the 1C donor SAM. Furthermore, we found increases in several enzymes involved in NAD^+^ metabolism: CG15093, a mitochondrial oxidoreductase that uses NAD^+^ as a coenzyme; Naxd, an epimerase that catalyses the ATP-dependent repair of chirally inactive forms of NADH and NADPH; and Gale, an NAD^+^-dependent epimerase involved in glucose metabolism [19].

We conclude that the expression of toxic Aβ-Arc in adult flies alters NAD^+^ metabolism by predominantly affecting mitochondrial complex I and 1C metabolism.

### Neuronal expression of Aβ-Arc reduces complex I-dependent NADH oxidation and results in reduced folate levels

**Fig. 2:**
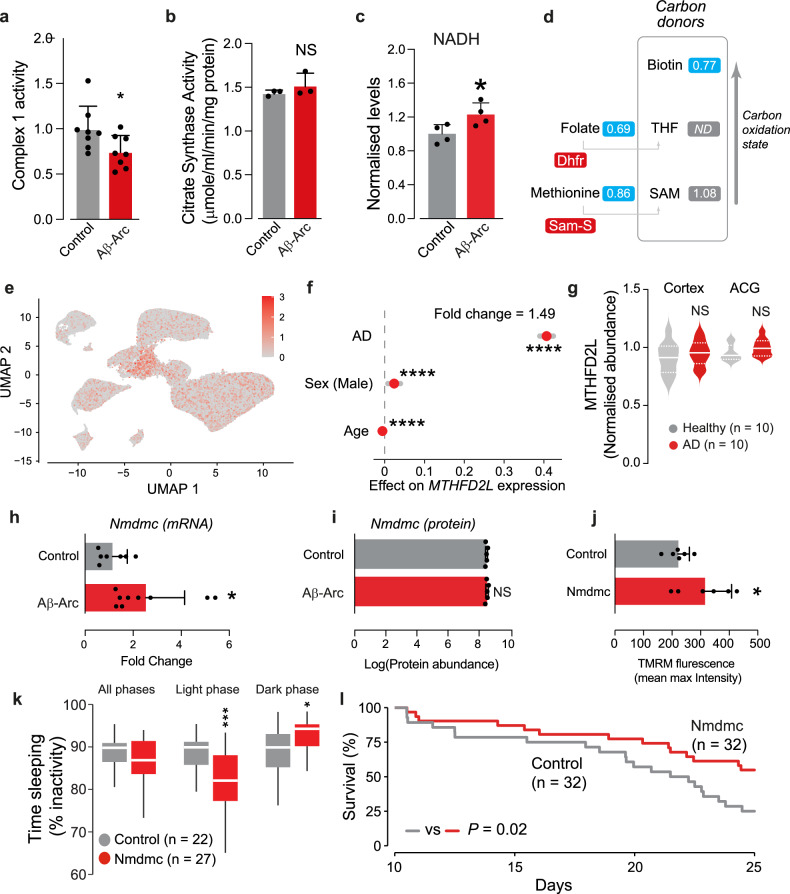
Expression of Aβ-Arc decreases mitochondrial complex I activity and folate levels. **a** Mitochondrial complex I activity is decreased in flies expressing toxic Aβ-Arc (means ± SDs; asterisks, two-tailed Student’s *t* test). **b** Expression of Aβ-Arc does not affect overall mitochondrial mass. Mitochondrial mass was assessed by measuring the activity of the mitochondrial matrix enzyme citrate synthase in adults (NS, *P* > 0.05, two-tailed unpaired *t* test compared to control). **c** Increased NADH levels in the heads of Aβ-expressing flies (means ± SDs; asterisks, two-tailed Student’s *t* test). **d** Fold-changes in metabolite abundance upon expression of Aβ-Arc in flies. Blue corresponds to metabolites that were significantly downregulated. ND corresponds to a metabolite below the detection threshold. Enzymes that were upregulated in Aβ-Arc-expressing flies are shown in red. The metabolite levels are shown to the right of each individual chemical entity. The statistical significance was determined using Welch’s two-sample *t*-test (*n* = 8). **e** Analysis of *MTHFD2L* expression levels in clustered neurons from both AD patients and controls. Cells are represented by dots and the expression level of *MTHFD2L* is represented as a two-colour heatmap. Data were obtained from a study by Otero-Garcia and colleagues [25]. **f** Neurons derived from AD patients have a higher expression of *MTHFD2L* (β = 0.37, standard error = 0.12, *P* = 0.008, linear regression). Supplementary analysis with additional covariates and a linear mixed model accounting for the different patients were performed (see GitHub repository: https://m1gus.github.io/AD-FA/). **g** Analysis of MTHFD2L protein levels in the brains of AD patients (median with interquartile range; asterisks, two-tailed Student’s *t* test). Data were obtained from a proteomics study on AD patients [75]. **h** Upregulation of the *Nmdmc* transcript in the heads of Aβ-expressing flies (means ± SD; asterisks, two-tailed Student’s *t* test). **i** Levels of Nmdmc protein are not altered in Aβ-expressing flies. Protein levels were measured by mass spectrometry (means ± SD; asterisks, two-tailed Student’s *t*-test). Neuronal expression of Nmdmc in adult flies increases Δψm in the brain (**j**, means ± SD; asterisks, two-tailed unpaired *t* test); alters the percentage of time spent asleep during the light and dark phases (**k**, asterisks, two-tailed unpaired *t* test) and increases the lifespan of adult flies (**l**, log-rank, Mantel–Cox test). Genotype: (**a,** **b,** **c,** **h,** **j,** **k** and **l**) *elavGal4; +;+* (Control), *elavGal4; UAS Aβ42Arc/+;* + (Aβ-Arc) and *elavGal4; UAS-Nmdmc/+ ;+* (Nmdmc) ; (**d** and **i**) *w; +; daGal4/+* (Control)*, w; UAS Aβ42Arc/+; daGal4/+* (Aβ-Arc).

Since we detected increases in several of the subunits of mitochondrial complex I in flies expressing toxic Aβ-Arc, we next measured complex I activity in their brains using an enzymatic assay that measures complex I-dependent NADH oxidation. We found that the ability of complex I to oxidise NADH was significantly reduced in flies expressing Aβ-Arc in their brains (Fig. 2a) when accounting for mitochondrial mass (Fig. 2b). This reduction, in turn, was associated with increases in the total levels of NADH in the heads of Aβ-Arc-expressing flies (Fig. 2c).

Aβ-Arc-expressing flies show mitochondrial impairment [15], and the folate pathway of 1C metabolism is neuroprotective in other fly models of neurodegeneration [20]. As we detected upregulation of several enzymes involved in 1C metabolism in Aβ-Arc-expressing flies, we next compared the levels of several metabolites involved in this pathway between controls and Aβ-Arc-expressing flies (Fig. 2d). We found reduced levels of biotin, as well as the two precursors for the synthesis of THF and SAM, folate and methionine, respectively (Fig. 2d). Folate and methionine act as substrates for the enzymes that are upregulated in Aβ-Arc-expressing flies, namely, Dhfr, which converts folate to THF, and Sam-S, which uses methionine and ATP to generate SAM. We conclude that Aβ toxicity in flies causes both a decrease in mitochondrial complex I function and reprogramming of 1C metabolism characterised by increased levels of enzymes and decreased levels of the metabolite.

### Upregulating Nmdmc improves healthspan in flies

Nmdmc, a component of mitochondrial 1C metabolism, acts downstream of Dhfr to reduce NAD(P)^+^ to NAD(P)H. We previously showed that the Nmdmc transcript is induced upon ER stress but its translation is blocked by the unfolded protein response (UPR) [20, 21]. UPR markers are widely present in the brains of AD patients (reviewed in ref. [22]). We therefore assessed the levels of the Nmdmc transcript as well as the translated protein in AD patients and fruit flies expressing Aβ-Arc.

The human orthologues of *Nmdmc* are *MTHFD2* and *MTHFD2L* (reviewed in ref. [16]). In mammalian cells, mitochondrial folate metabolism involves the activities of MTHFD2 and its isoform MTHFD2L [23]. *MTHFD2* is expressed during embryonic development but not in adult cells [24]. *MTHFD2L* is expressed at high levels in adult organs such as the brain [23] and is the functional mitochondrial methyl-THF dehydrogenase in differentiated cells (reviewed in ref. [16]).

We therefore interrogated previously published dataset to evaluate the expression of *MTHFD2L* at the single-cell level in the brains of AD patients and controls [25]. We found a higher expression of *MTHFD2L* expression in neurons from AD patients (Fig. 2e, f). However, the protein levels of MTHFD2L were not significantly altered in the brains of AD patients (Fig. 2g). We also observed that Aβ-Arc-expressing flies had higher transcript levels of Nmdmc (Fig. 2h) but the protein levels of Nmdmc remained unaltered (Fig. 2i). Taken together, data from AD patients and a fly model of AD showed that the transcriptional upregulation of Nmdmc or MTHFD2L does not result in higher protein levels.

We therefore next investigated the consequences of increasing the levels of Nmdmc in flies. The overexpression of Nmdmc increased mitochondrial membrane potential (Δψm, Fig. 2j), altered sleep duration (Fig. 2k) and increased lifespan (Fig. 2l). Taken together, our data show that higher levels of Nmdmc improve healthspan in flies.

### Targeted neuronal expression of *Nmdmc* rescues neurodegeneration in fly models of AD

**Fig. 3:**
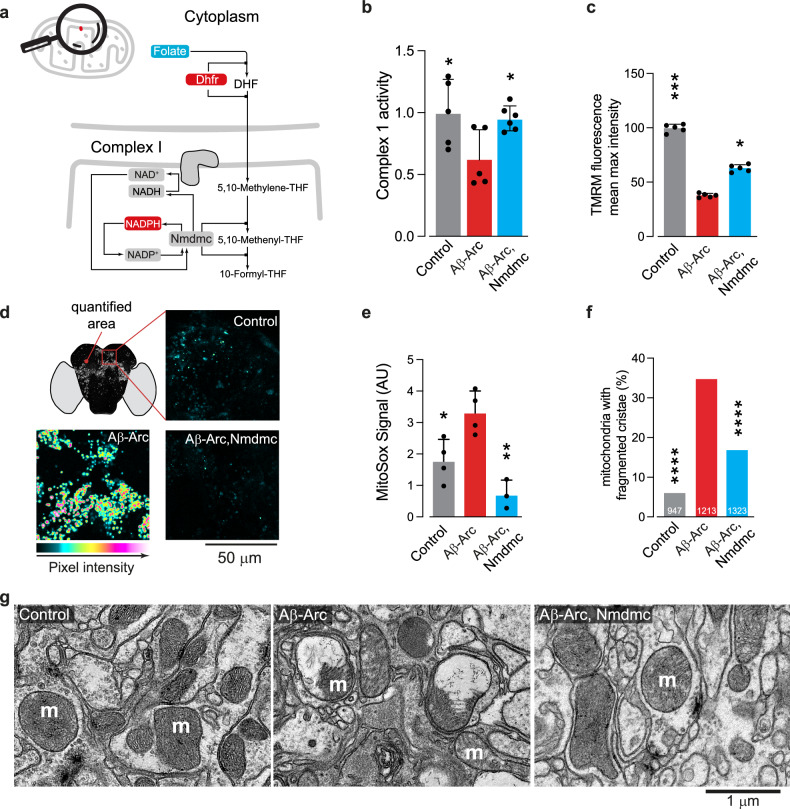
Mitochondrial defects caused by Aβ toxicity are reduced by *Nmdmc.* **a** Mitochondrial 1C folate metabolism. Dhfr reduces folate to dihydrofolic acid (DHF) and subsequently to THF. The NAD-dependent methylenetetrahydrofolate dehydrogenase (Nmdmc) protein converts 5,10-methylene-THF to 5,10-methenyl-THF and 10-formyl-THF in 2 steps, reducing the cofactor nicotinamide adenine dinucleotide phosphate (NAD(P)^+^) to NAD(P)H. Mitochondrial complex I oxidises NADH to NAD^+^ to translocate protons. For simplicity, some intermediate metabolites and enzymes are missing from this illustration. Red and blue correspond to metabolites or proteins with levels that were, respectively, significantly increased or decreased. Subunits of the mitochondrial complex I are shown as circles and follow the same colour convention. **b** Neuronal expression of *Nmdmc* improves complex I function in flies expressing Aβ-Arc (mean ± SD; asterisks, one-way ANOVA with Dunnett’s multiple comparison test, comparisons were relative to Aβ-Arc). **c** Improvement of Δψm in the brain following the expression of *Nmdmc* in Aβ-Arc-expressing flies (mean ± SD; asterisks, one-way ANOVA with Dunnett’s multiple comparison test, comparisons were relative to Aβ-Arc). The increase in mitochondrial ROS in the brains of Aβ-Arc flies is suppressed by the expression of Nmdmc. Representative confocal images (**d**) and quantitative analysis (**e**) of mitochondrial ROS using the MitoSOX Red fluorescent indicator in the indicated genotypes are shown (means ± SDs; asterisks, one-way ANOVA with Dunnett’s multiple comparison test, dots correspond to biological replicates i.e., brains). The intensity levels are visualised using a five-tone heatmap. **f**, **g** ultrastructural analysis of adult brains in control and Aβ-Arc expressing flies showing mitochondria with fragmented cristae in neuropiles (m, mitochondria) caused by the expression of Aβ-Arc. Representative TEM micrographs of the indicated genotypes are shown (**g**). **f** Percentages of mitochondria in the neuropiles that exhibited fragmented cristae (asterisks, two-tailed chi-square test, 95% confidence interval). Genotype: (**b**–**g**) Genotypes: *elavGal4; +;* + (Control)*, elavGal4; +; UAS Aβ42Arc/+* (Aβ-Arc)*, elavGal4; UAS Nmdmc/+; UAS Aβ42Arc/+ (*Aβ-Arc, Nmdmc).

THF acts as a donor of a carbon unit in the 1C pathway following the two-step reduction of folate to THF by Dhfr. In mitochondrial 1C metabolism, Nmdmc acts downstream of Dhfr in oxidation reactions that are thought to be coupled to complex I function (Fig. 3a).

Since overexpressing Nmdmc improved mitochondrial function in flies and given that Aβ-Arc-expression in flies causes mitochondrial dysfunction, we hypothesised that upregulating Nmdmc could protect against mitochondrial toxicity in fly models of AD. We found that neuronal expression of Nmdmc in flies expressing Aβ-Arc resulted in an increase in complex I activity (Fig. 3b) and recovery of Δψm (Fig. 3c). Methionine levels were also reduced in Aβ-Arc-expressing flies (Fig. 2d). The methionine cycle uses methionine as a substrate for SAM-dependent methyltransferases in the generation of the antioxidant glutathione. Complex I is a major site for ROS production, and impairment of complex I activity can increase ROS levels in mitochondria. Since both methionine levels and complex I activity were reduced, we measured mitochondrial ROS levels in the brains of flies expressing Aβ-Arc and detected increased levels (Fig. 3d, e). This increase in ROS was suppressed upon expression of Nmdmc (Fig. 3d). We previously showed that flies expressing Aβ-Arc have defects in mitochondrial morphology [15]. We next performed ultrastructural analysis by transmission electron microscopy (TEM) of mitochondria in the brains of adult flies. We observed that the overexpression of Nmdmc alleviated mitochondrial defects, reducing the percentage of fragmented cristae (Fig. 3f, g). Together, these data indicate that targeting the expression of Nmdmc to neurons restores mitochondrial health caused by Aβ-Arc toxicity.

**Fig. 4:**
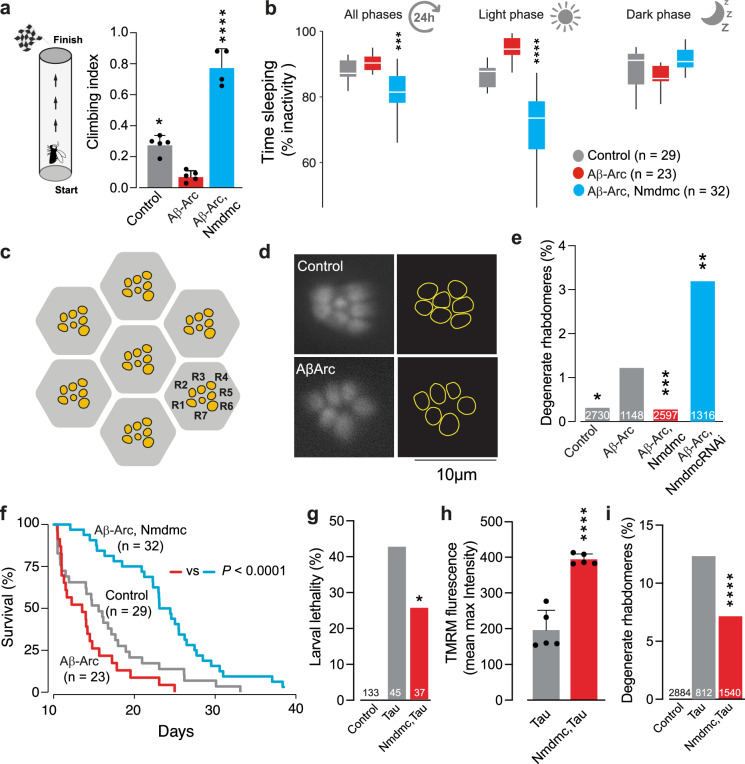
Neurodegeneration in fly models of AD is reduced by Nmdmc. **a** Flies expressing Aβ-Arc exhibited motor impairment. Expression of *Nmdmc* suppresses this locomotor defect (asterisks, one-way ANOVA with Dunnett’s multiple comparison test). **b** Expression of *Nmdmc* rescues the total percentage of time spent asleep, as well as sleep during the light phase in Aβ-Arc-expressing flies (asterisks, pairwise *t* test with the Benjamini‒Hochberg correction for multiple comparison). **c** An illustration of the typical layout of the visible photoreceptors (yellow, R1–R7) at the surface of the adult *Drosophila* ommatidia (grey hexagon). **d** Pseudopupil images from control and Aβ-Arc expressing flies. In the control ommatidia, seven rhabdomeres are visible (illustrated as yellow circles in the panel on the right). Neurodegeneration in Aβ-Arc resulted in ommatidia with a lower number of visible rhabdomeres. **e** Quantitation of the percentage of neurodegeneration of the photoreceptor cells in Aβ-expressing flies with up- or downregulation of *Nmdmc* (asterisks, two-tailed chi-square test, 95% confidence interval). **f** Neuronal expression of *Nmdmc* increases the lifespan of Aβ-Arc-expressing flies (log-rank, Mantel–Cox test). Increasing Nmdmc levels in tau-expressing flies reduces larval lethality (**g**, asterisks, two-tailed chi-square test, 95% confidence interval), improves Δψm (**h**, means ± SDs; asterisks, two-tailed unpaired *t* test) and decreases neurodegeneration (**i**, asterisks, two-tailed chi-square test, 95% confidence interval). Genotypes: *elavGal4; +;* + (Control)*, elavGal4; +; UAS Aβ42Arc/+* (Aβ-Arc)*, elavGal4; UAS Nmdmc/+; UAS Aβ42Arc/+ (*Aβ-Arc, Nmdmc), *elavGal4; +; UAS Tau0N4R/+* (Tau) and *elavGal4; UAS Nmdmc/+; UAS Tau0N4R/+* (Nmdmc,Tau).

In flies, Aβ toxicity disrupts neuronal activity and the sleep patterns of adult flies [26]. We observed that the increased neuronal expression of Nmdmc improved motor performance (Fig. 4a), decreased excessive sleep (Fig. 4b), reduced neurodegeneration of rhabdomeres (Fig. 4c–e) and improved the survival (Fig. 4f) of flies expressing toxic Aβ-Arc. Conversely, suppressing the expression of Nmdmc increased neurodegeneration in flies expressing toxic Aβ-Arc (Fig. 4e). Next, we tested if increasing the expression of Nmdmc could also rescue phenotypes associated with AD-associated tauopathy, caused by the expression of a toxic form of the microtubule-associated protein tau (0N4R tau). Expression of tau in flies causes larval lethality, which is decreased by Nmdmc overexpression (Fig. 4g). Similarly, Nmdmc overexpression in tau-expressing flies also improves Δψm in the brain (Fig. 4h) and decreases neurodegeneration (Fig. 4i). We conclude that neuronal expression of Nmdmc rescues mitochondrial dysfunction and is neuroprotective in fly models of AD.

### Mendelian randomisation analysis supports a protective role of *MTHFD2L* on AD risk

**Fig. 5:**
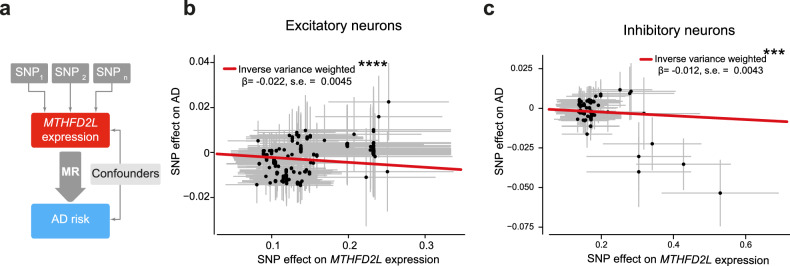
Higher *MTHFD2L* levels in neurons decreases AD risk. **a** MR uses SNPs to infer the causal effect of *MTHFD2L* expression on AD risk. MR uses genetic variants (SNPs in grey) that are associated with an exposure (*MTHFD2L* in red) as instrumental variables to evaluate the causal effect of that exposure on an outcome (AD risk in blue). Mendelian randomisation shows that higher *MTHFD2L* expression decreases AD risk in excitatory (**b**, β = −0.022, standard error = 0.0045, *P* < 0.00001, 337 SNPs, inverse variance weighted model) and inhibitory neurons (**c**, β = −0.012, standard error = 0.0042, *P* = 0.006, 154 SNPs, inverse variance weighted model).

Because the overexpression of *Nmdmc* protected flies against Aβ-Arc toxicity, we next explored whether genetic variations associated with the expression of *MTHFD2L*, the human orthologue of fly Nmdmc, are linked to AD severity in humans. Mendelia randomisation (MR) is a method that uses instrumental variables like SNPs to determine whether an exposure causes an outcome (reviewed in [27]), akin to a clinical trial (reviewed in ref. [28]). We thus used MR to investigate whether higher mRNA levels of *MTHFD2L* moderates AD risk (Fig. 5a). We found that higher *MTHFD2L* expression in both excitatory and inhibitory neurons decreases AD risk (Fig. 5b, c). Taken together, these results support the observation that elevated expression of *MTHFD2L* could be protective against AD pathology.

### Folinic acid restores mitochondrial health in AD models

**Fig. 6:**
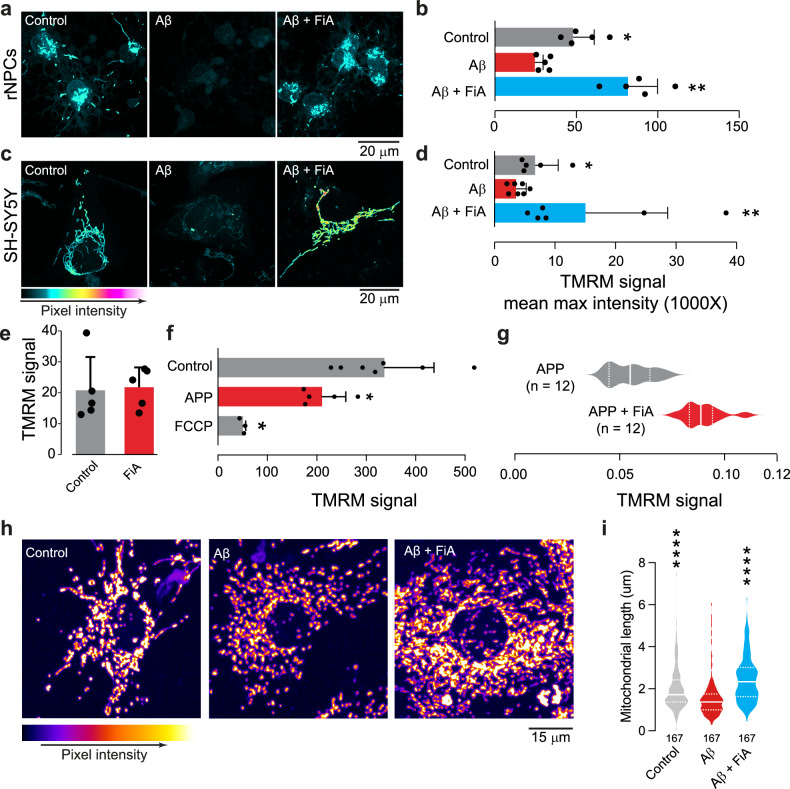
FiA improves mitochondrial function in cellular models of AD. Incubation of rat primary neurons (**a**, **b**) or human neuroblastoma cells (**c**, **d**) with FiA blocks the loss of mitochondrial Δψm caused by Aβ1-42 oligomers. Representative immunofluorescence images of 2 independent experiments (**a**, **c**) and quantitative analysis of TMRM fluorescence (**b**, **d**) in cells treated with the indicated agents. The intensity levels are visualised using a five-tone heatmap (mean ± SD; asterisks, one-way ANOVA with Dunnett’s multiple comparison test for **b** and pairwise Mann‒Whitney test with false discovery rate correction due to non normal distribution for **d**). **e** Addition of FiA in rat primary neurons does not alter TMRM levels (means ± SDs; asterisks, two-tailed unpaired *t* test). Cells were treated with 300 µM of FiA for 3 days. **f** SH-SY5Y cells over-expressing APPswe have lower levels of Δψm (mean ± SD; asterisks, one-way ANOVA with Dunnett’s multiple comparison test). **g** Addition of FiA increases Δψm in SH-SY5Y cells over-expressing APPswe (means ± SDs; asterisks, two-tailed unpaired *t* test). **h**, **i** Incubation of rat primary neurons with Aβ1-42 oligomers reduces mitochondrial length and this is reduced by FiA. Representative images of cells stained with anti-TOMM20 (**h**) and quantification of mitochondrial length (**i**). The total number (*n*) of analysed mitochondria is indicated (median with IQR; asterisks, Friedman test due to non-normal distribution of the data determined using the Shapiro–Wilk test).

Folinic acid (FiA) is a reduced form of folate that can cross the blood‒brain barrier [29]. Since we found that genetic enhancement of a mitochondrial component of the folate 1C pathway improves mitochondrial function in an AD model (Fig. 3), we next tested if an exogenous supplementation with FiA could also improve mitochondrial function in mammalian models of AD linked to Aβ toxicity. We treated either a differentiated human neuroblastoma cell line (SH-SY5Y) or primary rat neuronal progenitor cells (rNPCs) with Aβ1-42 oligomers for 48 hours and observed that the oligomers caused a loss of Δψm. Coincubation with both Aβ1-42 oligomers and FiA (Fig. 6a–d) prevented the loss of Δψm caused by the presence of Aβ1-42 oligomers. We did not find any alterations in Δψm in rNPCs incubated with FiA without Aβ1-42 oligomers (Fig. 6e). Next, we used SH-SY5Y cells stably expressing APP with the Swedish mutation (APPswe) [30]. We found that SH-SY5Y cells expressing APPswe have a decreased Δψm (Fig. 6f), which is recovered upon FiA supplementation (Fig. 6g). Finally, we sought to measure structural markers of mitochondrial dysfunction. We found that the addition of Aβ1-42 oligomers to cells in culture decreased mitochondrial length, which was suppressed by co-incubation with FiA (Fig. 6h, i). These results indicate that FiA supplementation improves mitochondrial health in cellular models of AD.

**Fig. 7:**
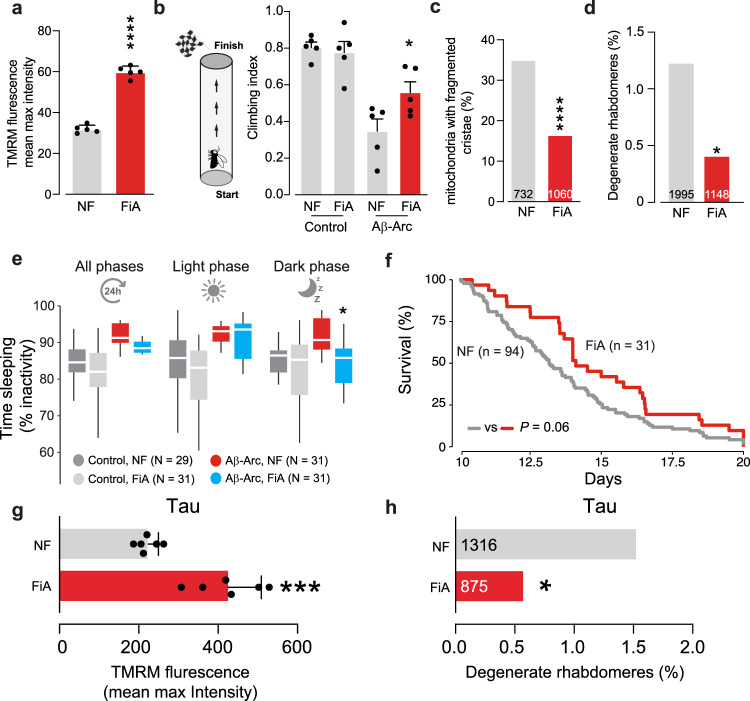
FiA restores mitochondrial health and is neuroprotective in fly models of AD. An FiA-supplemented diet in flies expressing Aβ-Arc improves Δψm in the brains (**a**, means ± SDs; asterisks, two-tailed unpaired *t* test); improves climbing abilities (**b**, asterisks, one-way ANOVA with Tukey’s multiple comparison test); reduces the degree of mitochondria cristae fragmentation (**c**, asterisk, two-tailed chi-square test, 95% confidence interval); reduces the neurodegeneration of photoreceptor cells (**d**, asterisks, two-tailed chi-square test, 95% confidence interval); rescues the percentage of time spent asleep during the dark phase (**e**, asterisks, pairwise *t* test with the Benjamini‒Hochberg correction for multiple comparison) and increases lifespan (**f**, log-rank, Mantel–Cox test). An FiA-supplemented diet in flies expressing Tau improves Δψm in the brains (**g**, means ± SDs; asterisks, two-tailed unpaired *t* test) and decreases neurodegeneration (**h**, asterisks, two-tailed chi-square test, 95% confidence interval). Genotype: *elavGal4; +;+* (Control), *elavGal4; UAS Aβ42Arc/+;* + (Aβ-Arc), *elavGal4; +; UAS Tau2N4R/+* (Tau).

We next tested if a FiA-supplemented diet could be neuroprotective in fly models of AD. Exogenous supplementation of Aβ-Arc-expressing flies with FiA improved mitochondrial function (Fig. 7a), increased climbing performance (Fig. 7b), decreased mitochondrial structural defects (Fig. 7c), reduced neurodegeneration (Fig. 7d), increased wakefulness (Fig. 7e) and lifespan (Fig. 7f). We also tested whether FiA could also improve neuronal health in flies expressing tau (2N4R). We found that feeding FiA to tau-expressing flies improved Δψm (Fig. 7g) and alleviated neurodegeneration (Fig. 7h). We conclude that a diet enriched with FiA restores mitochondrial health and is neuroprotective in fly models of AD.

### Folate intake is linked to decreased odds of developing AD

With age, the risk of developing AD increases and this is associated with decreased blood folate levels (reviewed in [31]). Alterations in the dietary intake of folate and in folate 1 C metabolism are also linked to AD (reviewed in ref. [32]), but the causal association between folate deficiency and AD is unknown.

**Fig. 8:**
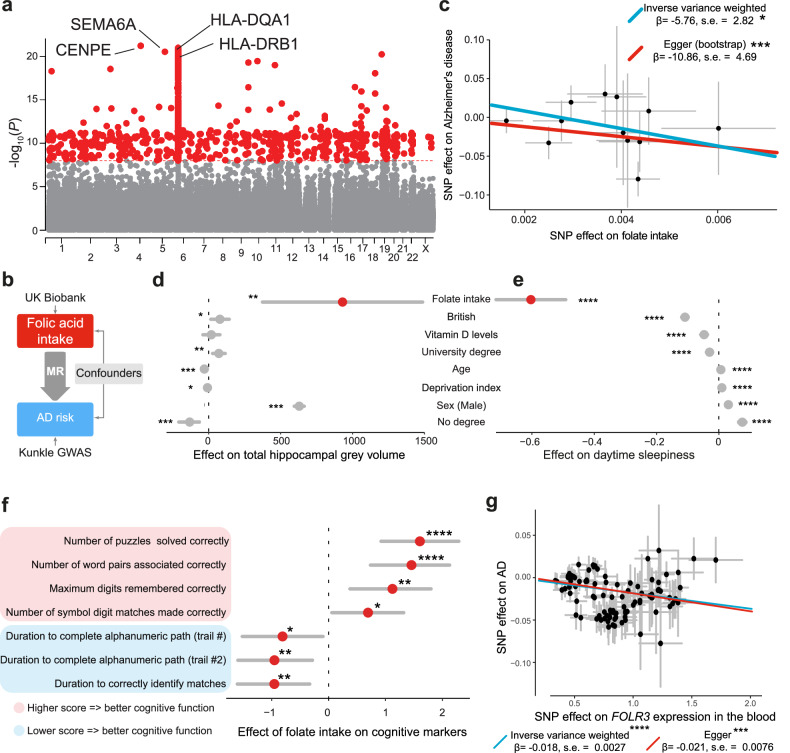
Higher folate intake is linked to decreased AD risk and pathologies. **a** Genome-wide association on folate intake in the UK Biobank. The source analysis was obtained from the Pan-UKB repository (https://pan.ukbb.broadinstitute.org). Genome-wide significant SNPs at a *P* value smaller than 10^−8^ are labelled in red and the rest in grey. Chromosomes are on the x axis. The top genes are labelled and the SNP localisation information was obtained from SNPdb [76]. **b** MR uses SNPs to infer the causal effect of folate intake on AD risk. MR uses genetic variants that are associated with an exposure (folate in red) as instrumental variables to evaluate the causal effect of that exposure on an outcome (AD risk in blue). MR is less prone to bias and confounding factors (in grey) than observational studies and can provide more robust evidence about causality. The origin of the genetic instruments are indicated beside each latent variable without boxes. We used genetic variants associated with the intake of folate from the UK Biobank and SNPs associated with AD risk from a GWAS conducted by Kunkle et al., 2019 to evaluate the causal relationship between the nutrient and AD. **c** Folate intake is causally associated with a decreased risk of developing AD (scaled estimate [95% CIs], asterisks, inverse variance weighted in blue and Egger regression in red). The error bars in grey show the standard error of the estimated effect (black dot) between the SNP on the exposure and the SNP on the outcome. SNPs related to folate intake were estimated based on the UK Biobank data, and AD-related SNPs were obtained from published data [71]. Folate intake is causally associated with higher hippocampal grey volume (**d**), decreased daytime sleepiness (**e**) and improved markers of cognitive function (**f**) (scaled estimate [95% CIs], asterisks, two-stage least square regression). The error bars in grey show the 95% CIs of the estimated effect (red dot). The investigated variable is in red. **g** Higher expression of the Folate receptor 3 (FOLR3) gene is causally associated with a decreased risk of developing AD (scaled estimate [95% CIs], asterisks, inverse variance weighted in blue and Egger regression in red). The error bars in grey show the standard error of the estimated effect (black dot) between the SNP on the exposure and the SNP on the outcome.

MR uses genetic data as instrumental variables to evaluate the causal relationship between an exposure (such as a particular diet, environmental factor, or medication) and an outcome (such as a disease or trait) [33]. Using data from the UK Biobank, we found genetic markers linked to folate intake (Fig. 8a). We next used MR to investigate the effect of folate intake on AD (Fig. 8b). We employed the inverse-variance weighted method, which combines the ratio estimates of the exposure and response variables, weighted based on their variance. Because this method can lead to bias in the presence of horizontal pleiotropy, we also used Egger regression to account for this bias. By combining both MR methods in our analysis, we found a link between folate intake and a reduced risk for AD (Fig. 8c). Next, we investigated whether dietary folate intake is linked to reduced AD pathologies that are correlated with disease severity [34]. Two-stage least squares (2SLS) models can be used to assess the effect of a specific variable (for example, folate intake) on continuous variables such as brain volume using instrumental variables (genetic data) and are commonly used in one-sample MR [35, 36]. Using this approach, we found that folate intake is linked to an increased hippocampal grey volume (Fig. 8d), reduced daytime sleepiness (Fig. 8e) and improvements in multiple cognitive markers for AD (Fig. 8f). Finally, tested the validity of our findings by assessing the connection between folate metabolism and AD risk in independent cohorts with different analytical methods. We indirectly modelled folate intake by using genetic loci associated with the expression of FOLR3, which is regulates uptake of folate [37, 38]. Using MR, we found that higher expression of FOLR3 is linked to a decreased risk of developing AD (Fig. 8g). Together, our findings indicate that folate intake could be beneficial for reducing AD risk.

## Discussion

Proteomic analysis of flies expressing Aβ-Arc has shown that the expression of most subunits of mitochondrial complex I is increased, while the activity of the complex is decreased. The relationship between these two findings is unclear. Aβ can undergo mitochondrial import via the translocase of the outer membrane (TOM) machinery [39]. It is therefore possible that Aβ-Arc can impair the mitochondrial import machinery and cause defects in the import and assembly of nuclear-encoded proteins such as subunits of complex I, leading to an uncoordinated assembly of subunits coded by the nuclear and mitochondrial genomes. Additionally, Aβ-Arc could lead to mitochondrial toxicity by preventing the correct assembly of complex I. As we found increases in the levels of complex I subunits, it is possible that Aβ-Arc can directly interact with subunits of complex I and prevent its correct assembly. Future research is needed to determine whether Aβ-Arc interacts directly with specific mitochondrial proteins and how that influences the correct assembly of complex I.

Inhibition of complex I has been linked to neurodegeneration. In particular, environmental exposure to inhibitors of complex I contributes to the pathogenesis of neurodegenerative disorders such as Parkinson’s disease (reviewed in [40]). Complex I catalyses the transfer of electrons from NADH to coenzyme Q10 and translocates protons across the inner mitochondrial membrane. It is unclear which function(s) of complex I—NADH oxidation, Q10 reduction and proton pumping activity are important in neuroprotection. Oxidation of NADH is sufficient to improve lifespan in *Drosophila* [41]. Future studies could test the relevance of the oxidoreductase activity of complex I as a neuroprotective strategy in *Drosophila* models of AD, for example, by expressing Ndi1, the yeast single-subunit NADH dehydrogenase in flies expressing Aβ-Arc.

We detected elevated levels of Sam-S and reduced levels of methionine, a substrate for Sam-S, in flies expressing Aβ-Arc (Fig. 2d). However, we found no changes in the levels of SAM. We thus hypothesise that there is an increased use of SAM. Mitochondrial SAM is required for the methylation and assembly of complex I [42], so we reasoned that the upregulation of Sam-S could represent an attempt to compensate for the complex I dysfunction caused by the expression of Aβ-Arc. Moreover, these data were collected from whole flies in a previous study. Future work can leverage technical advances in mass spectrometry and validate the changes in these metabolites in neurons.

Biotin is a dietary vitamin required by humans that transfers activated carboxyl groups. Biotin is abundant in many foods and synthesised by intestinal bacteria. We found that expression of Aβ-Arc led to decreases in the levels of this 1C donor (Fig. 2d). The citric acid cycle in the mitochondria of eukaryotes comprises several sequential reactions, including two decarboxylation reactions, that convert citrate to oxaloacetate, releasing two CO_2_ molecules. The citric acid cycle also consists of anaplerotic reactions catalysed by pyruvate carboxylase, phosphoenolpyruvate carboxykinase, phosphoenolpyruvate carboxylase and malic enzyme. The enzymes that catalyse carboxylation reactions usually employ biotin to activate CO_2_ and to carry it to acceptors such as pyruvate or phosphoenolpyruvate. It is conceivable that the decrease in biotin observed in Aβ-Arc-expressing flies compromised the anaplerotic reactions in mitochondria, further contributing to mitochondrial dysfunction. This could have also been the consequence of gut dysfunction in these flies. Further work is required to determine whether the neuronal expression of Aβ-Arc in our fly model caused gut dysfunction.

Disrupted sleep is associated with AD. We and others have shown that patients diagnosed with AD report being more tired than controls [15]. We found that expression of Nmdmc in flies decreased sleep during the light phase in Aβ-Arc-expressing flies, which we reasoned to be due to the improvement in complex I activity. Complex I- and complex III-mediated ROS levels are higher during the light phase than during the dark phase. It is conceivable that the complex I impairment in our model exacerbated the increases in ROS levels during the light phase, thereby leading to increased sleep. Constitutive expression of Nmdmc restored complex I activity and could have alleviated the ROS-induced alteration of sleep patterns. In addition, NADP^+^ can bind to the oxidoreductase domain of the potassium channel Shaker and cause sleep-inducing tonic firing of neurons in the dorsal fan-shaped body (dFB) of the fly brain [43]. It is therefore possible that the oxidation of NADPH to NADP^+^ by Nmdmc promotes wakefulness by decreasing the availability of NADP^+^ to Shaker and reducing sleep-inducing neuronal activity. Future work could thus measure the spiking activity of dFB.

We also found that the overexpression of Nmdmc in flies (Figs. 3 and 4) led to the rescue of phenotypes (e.g. sleep duration in Fig. 4b and lifespan in Fig. 4f) that was stronger than control. The expression of Nmdmc in non-AD flies can also improve these markers (sleep duration in Fig. 2k and lifespan in Fig. 2l). Nucleotide metabolism comprises the de novo and the salvage branches to generate metabolites used for the synthesis of DNA, RNA and co-factors in enzymatic reactions. Nmdmc is part of the de novo branch and dNK is part of the salvage branch. We showed that dNK expression in flies also improved the overall fitness of adult flies [44]. These observations suggest that genetically enhancing mitochondrial nucleotide metabolism resulted in a strong rescue of certain phenotypes.

We observed that FiA supplementation caused flies to sleep less during the dark phase than during the light phase. This could have been due to feeding patterns. Flies tend to feed at the beginning of the light phase [45], and FiA might only become active in the brain in a few hours post-feeding. Addressing this question would require tracking the intake of FiA in adult flies during their daily cycle.

Fly models cannot fully recapitulate human diseases. Flies do not have an adaptive immune system, and the axons of fly neurons are not myelinated like those of mammals. Therefore, testing if enhancing 1C metabolism is neuroprotective in mammalian model organisms or clinically in patients is important. Our study aimed to establish the relevance of our results to humans using MR. MR showed that genetic variants in *MTHFD2L* associated with higher levels of its transcript are linked to a reduced AD risk. However, there are technical limitations that can decrease the robustness of MR analysis. For example, genetic polymorphisms can explain a fraction of the expression of *MTHFD2L* mRNA expression leading to weak instruments and breaking the NO Measurement Error assumption [46]. The absence of a mechanistic understanding of how each SNP affects *MTHFD2L* mRNA levels in neurons can also affect the reliability of our predictions (reviewed in ref. [47]). Future work is required to define how the SNPs used in the MR analyses control the expression levels of *MTHFD2L*. Nonetheless, we reason that our MR analyses are reliable as they independently replicate experimental observations.

Specifically, we found that, in AD, *MTHFD2L* expression is increased at the transcript but not protein levels, suggesting that there could be issues with translating this gene, possibly due to UPR. We also provide evidence that higher *MTHFD2L* transcript expression is linked to decreased AD. The predicted increase in *MTHFD2L* transcript expression might not be translated in the context of AD. We mitigate the potential of reverse causation, which is the effect of AD on *MTHFD2L* expression when analysing the effect of *MTHFD2L* on AD, by using MR [48]. Additionally, our analyses model a life-long exposure to higher *MTHFD2L* transcript levels on the subsequent odds of developing AD, which circumvents the impact of AD on *MTHFD2L* expression. The combination of Mendelian randomisation and mRNA expression analyses also allows us to distinguish between risk factors and adaptations as shown previously [49]. Specifically, our observations that higher *MTHFD2L* expression is protective against AD, as well as that the increased *MTHFD2L* expression at the single-cell level indicates that increased *MTHFD2L* mRNA levels is an adaptive response in AD. Future research work could investigate the effect of increasing *MTHFD2L* transcript levels via mRNA delivery on AD pathology in mammalian models.

Serum folate levels are decreased in patients with AD [50], but the causative relationship between folate intake and AD remains controversial [51, 52]. We found that increased folate intake reduces the risk for AD-related pathologies using MR (Fig. 8). A caveat of using MR to model behavioural traits is that the traits are often confounded or misreported [53]. However, we noted multiple SNPs linked to folate intake, which are above genome-wide significance, suggesting that the instrument is statistically robust. An additional method to investigate the effect of folate on AD risk would have been to use serum folate levels as an instrumental variable instead of folate intake. However, MR requires reliable genetic instruments associated with folate levels that are also linked to AD. These were not available for our study. Instead, we used the expression of FOLR3 in the blood as a genetic surrogate for folate intake. Previous research showed that increasing FOLR3 expression led to an increased folate concentration in cells [38, 54]. We showed that a higher folate intake by cells is also associated with decreased AD risk, which provides an independent validation of our previous analysis. A clinical trial on the effect of folate on mitochondrial function in the brain in AD patients would provide more robust evidence to supplement our observations.

Our data, obtained by combining an animal model of AD with information from AD patients, suggest that enhancement of mitochondrial folate 1C metabolism is a viable strategy for neuroprotection in AD. We propose that this enhancement can be combined with other therapies to restore brain health in AD as well as other diseases associated with mitochondrial dysfunction (reviewed in [55]).

## Methods

### Genetics and *Drosophila* strains

Fly stocks and crosses were maintained on standard cornmeal agar media at 25 °C. The strains used were *elav*GAL4 (pan-neuronal driver), *w; UAS Aβ42ARC; +* and *w; 51D;* + (described in Yu et al., 2021), *da*Gal4 (Ubiquitous driver) and *UAS Tau2N4R* (Bloomington Drosophila Stock Center), *w; +; UAS Aβ42ARC and w; +; 86* *F*, (kind gifts from D. Crowther, R&D Neuroscience, Innovative Medicines and Early Development Biotech Unit, AstraZeneca, Cambridge, UK), *UAS_Nmdmc* and RNAi Nmdmc (described in Celardo et al., 2017) and *UAS Tau0N4R* (kind gift from J. Hodge, School of Physiology, Pharmacology and Neuroscience, University of Bristol, Bristol, UK). Male elav>Aβ-Arc and elav>Tau2N4R flies were used and female elav>Tau0N4R were used due to high larval lethality.

### Metabolic profiling

The analysis of the levels of methionine, biotin and SAM was obtained from global metabolic profiling using the Metabolon Platform (Metabolon Inc., NC, USA) and as described previously [15]. The metabolites were profiled in adult females expressing Toxic Aβ-Arc using the ubiquitous *daughterless* (*da*) Gal4 driver, aged for 15 days after eclosion. Each biological replicate comprised 100 15-day-old adult female flies (approximately 100 mg per replicate), with a total of 7–8 replicates per genotype.

Folate levels were measured using an enzymatic assay following the manufacturer’s protocol (BioVision, E4523-100). Two 10-day-old flies per sample were frozen using liquid nitrogen and homogenised in 100 µL of the provided Sample Diluent for 30 seconds. The folate levels were normalised to the total protein levels, which were measured using a Pierce BCA Protein Assay Kit (Thermo Fisher Scientific, 23227).

NADH concentrations were measured using the NAD + /NADH EnzyChrom colorimetric assay according to the manufacturer’s instructions (BioAssay Systems, CA, USA). Briefly, 10 fly heads were homogenised in the specified buffer on ice at zeitgeber-7. NADH concentrations were normalised to total protein measured using a bicinchoninic acid assay. The absorbance was measured using an Infinite M200Pro multifunction reader (TECAN, Mannedorf, Switzerland). All measurements were normalised to the average of the control samples (elav>51D).

### Cell culture

Human neuroblastoma cells (SH-SY5Y) were cultured in Dulbecco’s modified Eagle medium (DMEM; Thermo Fisher Scientific, Waltham, MA, USA, 41966) supplemented with 10% foetal bovine serum (FBS; Merck, Kenilworth, NJ, USA, F9665). All cell media were supplemented with 1% penicillin and streptomycin (Thermo Fisher Scientific, Waltham, MA, USA, 15070063). The cells tested negative for mycoplasma, and their identity was validated by STR genotyping.

The differentiation of the SH-SY5Y cells into mature neuron-like cells was performed as previously described [56]. Briefly, cells were treated with 10 µM all-trans retinoic acid (RA, cat. R2625, Sigma‒Aldrich, Bornem, Belgium) and 5% FBS in DMEM (cat. 41965-039, Invitrogen) for 3 days. The medium was then changed to Neurobasal-A medium (cat. 12349-015, Invitrogen) supplemented with 1% (v/v) l-glutamine (200 mM, cat. 25030-024, Invitrogen), 1% (v/v) N-2 supplement 100× (cat. 17502-048, Invitrogen) and 50 ng/mL BDNF (ab206642) for another 3 days. For drug treatment, Aβ1-42 oligomers (MP Cambridge Bioscience, AnaSpec 20276-ANA) were added to the cell medium at a 2 µM final concentration for 48 hours, and the medium was changed to medium containing 2 µM Aβ1-42 oligomers and 300 µM FiA for an additional 48 hours.

### Isolation and culture of rat neural progenitor cells

Rat neural progenitor cells were cultured as previously described [57] with the following modifications. Briefly, neonatal rat whole brains (7 days old) were minced and resuspended in 2 mL of enzymatic solution containing papain (34 u/mL; Worthington, #LS003127) and DNase (40 ug/mL; Sigma, #D5025) in Hibernate A Low-Fluorescence (HALF; Transnetyx Tissue; #HALF) and digested on an orbital shaker at 50 rpm and 35 °C for 40 minutes. At the end of the incubation time, the tissue suspension was mixed with 6 mL of Hank’s balanced salt solution (14175-053, Gibco) and centrifuged (300 × *g*, 2 min, room temperature [RT]). To yield a single-cell HBSS suspension, samples were gently triturated 10 times each in trituration solution (2 mM sodium pyruvate, 2% B27 in HALF) with a 5 mL stripette and 3 fire-polished pipettes of decreasing diameters. The cells were passed through a 70 µM cell strainer into a tube containing 90% isotonic Percoll (GE Healthcare, #17-0891-01) in 10X PBS (Gibco, #70013032). The final volume was achieved with DMEM/F12 (Gibco, #31331028) to yield a final Percoll concentration of 22.5%. The solution was mixed, and the single-cell suspension was then separated from tissue and myelin debris by gradient density centrifugation at 800 × *g* (without brakes) for 20 min at RT. After the spin, the myelin debris and supernatant were aspirated, leaving only the cell pellet, which was resuspended in HBSS to wash out the Percoll and then centrifuged (300 × *g*, 5 min, RT). The resulting cell pellet was resuspended in lysis buffer (1 mL, BD Biosciences, #555899) for 1 min to remove contaminating red blood cells. Following centrifugation (300 × *g*, 5 min, RT), the pellet was resuspended in Neurobasal medium (Gibco; #21103049) supplemented with B27 (1:25; Gibco #17504044), 1 mM GlutaMAX (Gibco, #35050061), penicillin‒streptomycin (Gibco, # 15140122), EGF (20 ng/mL, PeproTech, # 315-09) and bFGF (20 ng/mL, PeproTech, # 100-18B) and then plated at a density of 5 × 10^5^ viable cells/cm^2^. After overnight (16 h) incubation in a humidified chamber at 37 °C and 5% CO_2_, the adherent cells attached to the tissue culture plastic, while putative NPCs remained in suspension. The supernatant containing the NPCs was transferred to a new T25 flask and maintained in Neurobasal medium until floating clusters of proliferating cells started to appear in approximately 5–7 days. To propagate the cultures, the cell suspension was transferred into a fresh 15 mL sterile conical tube and pelleted (300 × *g*, 5 min, RT). Then, the pellet was dissociated by pipetting up and down (20–30x) with a P1000 pipette to produce a single cell suspension. Viable cells were then subpassaged at a density of 5 × 10^3^ cells/cm^2^ onto fresh uncoated plates. To induce differentiation into neurons, NPCs were pelleted as previously described, seeded at a density of 1 × 10^4^ cells/cm^2^ PDL-laminin-coated chambered coverglass (Thermo Scientific, 155411PK), and maintained in Neurobasal medium.

### Drug treatments

FiA was incorporated directly into the fly food at a final concentration of 4 mM. Crosses were set up on normal food and transferred to drug food after 2 days. Larvae were treated with the drug throughout development. The adult flies were kept on drug-containing food throughout their lifespan, and they were transferred to vials with fresh food every 3 days. Flies from each genotype were randomly assigned to normal food and supplemented food.

### Locomotor assays and lifespan analysis

The analyses of sleep and lifespan in flies were performed as described in [15]. Adult male flies were aged to 10 days post-eclosion and individually loaded in glass tubes containing the same food used for rearing. The flies were grown and analysed in a light/dark 12 h/12 h cycle at 25 °C. The total number of recorded midline crossings per minute was recorded using the Drosophila Activity Monitoring System (Trikinetics, Waltham, MA), and the data were analysed using Rethomics. The analysis started at the first ZT0 to allow acclimation. Sleep was defined as any period of uninterrupted behavioural immobility (0 counts per minute) lasting more than 5 min [58–60]. Sleep was calculated for the first 5 days, and the data of flies that died were discarded. One-way analysis of variance with Tukey’s multiple comparison test was used to determine significance for the fraction of time asleep. The data for lifespan analysis are presented as Kaplan–Meier survival distributions. We recorded the entire lifespan of the flies from 10 days post-eclosion until their death and determined statistical significance using the log-rank test. We provide the full analysis script and the raw data in our GitHub repository.

### Climbing analysis

Climbing assays were performed using a counter-current apparatus equipped with six chambers. The assay was performed in an undisturbed room at ZT 2, with consistent lighting and a heater to warm the room to approximately 25°C (confirmed via a thermometer) using a quiet portable heater. Approximately 12 male flies were placed into the first chamber, tapped to the bottom, and then given 10 seconds to climb a distance of 10 cm. The flies that successfully climbed 10 cm or beyond within the allocated time were then shifted to a new chamber, and both sets of flies were given another opportunity to climb the 10 cm distance. This procedure was repeated a total of five times. After five trials, the number of flies in each chamber was counted to calculate the climbing index. A video demonstrating this technique can be found at https://youtu.be/vmR6s_WAXgc. The climbing index was measured using a weighted average approach with the following formula:

$${Climbing\; index}=\frac{0\times {n}_{0}+1\times {n}_{1}+2\times {n}_{2}+3\times {n}_{3}+4\times {n}_{4}+5\times {n}_{5}\,}{{Total\; number\; of\; flies}* 5}$$

In this formula, ${n}_{0}$ to ${n}_{5}$ corresponds to the number of flies that were left in each of the 6 bottom tubes shown in the video (https://youtu.be/vmR6s_WAXgc). Flies that died during the experiment or caught in the apparatus were removed from counting.

### Pseudopupil analysis

The heads of 5-day-old flies were directly fixed on standard microscope slides using quick-dry transparent nail varnish as described [15]. A Zeiss Axioplan 2 microscope equipped with a 63x oil immersion objective was used to visualise the ommatidia. Around 5 flies per condition were examined, to obtain a total number of approximately 1000 rhabdomeres. The percent abnormal rhabdomeres was calculated as the number of degenerate rhabdomeres over the total number of rhabdomeres: (A*1 + B*2 + C*3) / N, where A = number of ommatidia with 6 rhabdomeres, B = number of ommatidia with 5 rhabdomeres, C = number of ommatidia with 4 rhabdomeres and N = total number of ommatidia counted. Statistical significance was determined using a chi-squared test.

### Microscopy-based assessment of mitochondrial function and length

Measurements of Δψm in fly brains were performed as previously described [44]. Briefly, fly brains were loaded for 40 min at room temperature with 40 nM TMRM in loading buffer (10 mM HEPES pH 7.35, 156 mM NaCl, 3 mM KCl, 2 mM MgSO_4_, 1.25 mM KH_2_PO_4_, 2 mM CaCl_2_, 10 mM glucose), and the dye was present during the experiment. In these experiments, TMRM was used in the redistribution mode to assess Δψm, and therefore, a reduction in TMRM fluorescence represented mitochondrial depolarisation. Confocal images were obtained using a Zeiss LSM 880 confocal microscope. The illumination intensity was kept to a minimum (at 0.1–0.2% of laser output) to avoid phototoxicity, and the pinhole was set to give an optical slice of 2 μm. Fluorescence was quantified by exciting TMRM using the 565 nm laser and measured above 580 nm. Z-stacks of 5 fields of 300 μm^2^ each per brain were acquired, and the mean maximal fluorescence intensity was measured for each group.

For measurements of Δψm in cultured cells, the cells were loaded with 25 nM TMRM for 20 min at 37 °C, and the dye was present during the experiment. The TMRM was measured using a Zeiss LSM 880 or 980 confocal microscope equipped with a 63x oil immersion lens at 37 °C.

For the ROS assays, the brains of 5-day-old male flies were dissected in cold PBS and incubated with 5 μM MitoSOX Red mitochondrial superoxide indicator (M36008, Molecular Probes) for 30 min. After incubation, the brains were washed with PBS for 10 min and immediately imaged on a Zeiss LSM880 confocal microscope. Eighty-nine-micrometre-thick stacks were acquired. The maximal intensity projections of the MitoSOX signal in the fly midbrain were quantified using ImageJ.

The mitochondrial length was measured in primary cultured cells. The cells, NPCs pooled from two brains, were cultured in 24-well plates with glass bottom and imaged without bias on an Opera Phenix Plus High-Content Screening System (PerkinElmer Inc). Moreover, 20 μm z-stacks were collected and projected. The mitochondrial length was measured manually across the largest dimension of each mitochondrion using ImageJ. One image from each well was selected at random and around 40 random measurements were taken per image.

### Transmission electron microscopy

For transmission electron microscopy, adult fly brains were fixed for 2 h in 0.1 M sodium cacodylate buffer (pH 7.4) containing 2% paraformaldehyde and 2.5% glutaraldehyde (at room temperature). The samples were subsequently fixed for 1 h at room temperature in a solution containing 1% osmium tetroxide and 1% potassium ferrocyanide. After fixation, the samples were stained *en bloc* with 5% aqueous uranyl acetate overnight at room temperature. The sample were dehydrated via a series of ethanol washes and embedded in TAAB epoxy resin (TAAB Laboratories Equipment Ltd., Aldermaston, UK). Semithin sections were stained with toluidine blue, and areas of the sections were selected for ultramicrotomy. Ultrathin sections were stained with lead citrate and imaged using a TemCam XF416 digital camera and EM Menu software (TVIPS, Gilching, Germany) with a Joel 1400 electron microscope (Joel UK Ltd., Welwyn Garden City, UK). At least three flies per genotype or condition were analysed.

### Measurement of mitochondrial complex I activity

Ten flies per sample were flash frozen, and their heads were isolated. Mitochondria were extracted by homogenising the fly heads in a Dounce homogeniser using a tight pestle (Stretton Scientific, P000933LYSK0A.0) in 100 µL of PBS with 1x EDTA-free cOmplete™ protease inhibitor cocktail (Roche). To extract crude mitochondria, the samples were centrifuged at 600 × *g*, and the supernatant was subsequently centrifuged at 12,000 × *g*, both at 4 °C. Complex 1 activity was assessed using a colorimetric ab109721 kit. Assays were conducted on 96-well microtiter plates using an Infinite M200Pro multifunction reader (TECAN, Mannedorf, Switzerland). Complex I activity levels were normalised to the total protein levels measured using a Pierce BCA Protein Assay Kit (Thermo Fisher Scientific, 23227).

### Citrate synthase assay

Thirty flies per sample were flash frozen, and their heads were isolated. Citrate synthase activity was measured using a protocol adapted from the Citrate Synthase Assay kit (CS070 SIGMA) and described previously [61]. Briefly, heads from 30 flies were homogenised in lysis buffer (100 mM KCl, 20 mM Hepes at pH 7.5, 5% (v/v) glycerol, 10 mM EDTA, 0.1% (v/v) Triton X-100, 10 mM DTT, 1 μg/mL leupeptin, 1 μg/mL antipain, 1 μg/mL chymostatin and 1 μg/mL pepstatin). The suspensions were cleared twice by centrifugation at 2000 × g for 15 s at 4 °C, and the protein concentrations were determined by Bradford assay (Bio-Rad). Sample volume was resuspended in reaction buffer (75 mM Tris-HCl pH 8, 100 μM DTNB, 0.1% Triton, 350 μg/ml, 0.5 mM Oxalacetate) and absorbance was measured at 412 nm for 2 min using M200PRO plate reader (TECAN, Switzerland). Absorbance values were plotted against time (min) for each reaction. Changes in absorbance (ΔA412/min) were used to calculate the citrate synthase activity. Units of citrate synthase activity were normalised to protein concentration (mg/ml).

### RNA extraction and quantitative real-time PCR with reverse transcription

Total RNA was extracted from 30 heads per sample gut using TRIzol (Ambion) and quantified by spectrophotometric analysis (Nanodrop, Thermo Scientific). Quantitative real-time PCR with reverse transcription (RT–qPCR) was performed with a real-time cycler (Applied Biosystems 7500, Fast Real-Time PCR Systems) using a SensiFAST SYBR Lo-ROX One-Step Kit (Bioline). The fold change values were calculated using the comparative Ct method [62]. For RT–qPCR, we measured the coefficient of variation (CV) of the technical replicates and excluded from statistical analysis any samples with CV > 3%. The Nmdmc primer set was obtained from QIAGEN (QuantiTect Primer Assays, no. QT00503153). rp49 was used as a housekeeping gene: forward, 5′-TGTCCTTCCAGCTTCAAGATGACCATC-3′, reverse 5′-CTTGGGCTTGCGCCATTTGTG-3′.

### Proteome profiling

Proteomic analysis was performed as previously described [63]. Protein extracts from whole flies were prepared by grinding in radioimmunoprecipitation assay (RIPA) buffer (20 mM Tris pH 7.5, 150 mM NaCl, 1% [v/v] Nonidet P40, 0.5% [w/v] sodium deoxycholate and 1 mM EDTA) supplemented with 1 µg ml^–1^ leupeptin, 1 µg ml^–1^ antipain, 1 µg ml^–1^ chymostatin, and 1 µg ml^–1^ pepstatin and phosphatase inhibitor cocktail (PhosSTOP, Roche). Suspensions were cleared by centrifugation at 21,000 × g and 4 °C for 10 min, and the protein concentrations of the supernatants were measured using the Bradford assay (Bio-Rad). The cleared lysates were stored at −80 °C until required for proteomics analysis.

For proteomics analysis, the raw data files were processed using Proteome Discoverer v.2.1 (Thermo Fisher Scientific) and Mascot (Matrix Science) v.2.6. All comparative analyses were performed with R statistical language. The R package MSnbase was used for processing the proteomics data. Briefly, this process entailed the removal of missing values (instances where a protein was identified but not quantified in all channels were rejected from further analysis), log2-transformation of raw data and subsequent sample normalisation utilising the ‘diff.median’ method in MSnbase (this translates all sample columns such that they match the grand median). The differential abundances of proteins were evaluated using the limma package, with their variances moderated by the empirical Bayes method. *P* values were adjusted for multiple testing using the Benjamini–Hochberg method.

To select the top proteins differentially expressed in the Aβ-Arc group compared to the control group, we first performed a PCA [64] on the scaled abundances of all the significantly different proteins. PC1 accounted for most of the variance; therefore, we selected the proteins that contributed to PC1 and had a fold change larger than 1.5 or smaller than −1.5, resulting in 299 proteins. We used this list to query the STRING database [65], specifically focusing on UniProt keyword analysis. We also performed the same functional enrichment analysis using all proteins that were significantly changed at a false discovery rate of < 0.05 (1578 proteins). This is included as Supplementary data (github: https://m1gus.github.io/AD-FA/).

### UK Biobank analysis and MR

The UK Biobank comprises health data from over 500,000 community volunteers based in England, Scotland, and Wales. The geographical regions, recruitment and other characteristics has been previously described [66]. Informed consent was obtained from all subjects. UK Biobank ethical approval was granted by the Northwest Multi-Centre Research Ethics Committee. The current analysis was approved under UK Biobank application #60124. The variable used for AD diagnosis is derived from Category 47, Data-Field 42021 of the UK Biobank data showcase. Specifically, the UK Biobank Outcome Adjudication group generated an algorithm that links data from 3 sources, namely self-reported information from the UK Biobank, hospital-admission data and death register data [67], to achieve a high diagnostic accuracy.

Mendelian randomisation analyses were performed according to guidelines [68]. For one-sample Mendelian randomisation analysis, we used two-stage least square regressions using the UK Biobank cohort. Polygenic risk scores for folate intake were generated using PRSice [69]. For two-sample Mendelian randomisation, we used an inverse variance weighted and Egger regression using the TwoSampleMR package [70]. The GWAS statistics for folate intake in the UK Biobank were obtained from the Pan-UKB Team (https://pan.ukbb.broadinstitute.org). The genome-wide association study of AD was obtained from a published study [71] as the second sample. Forest plots were generated using yytools (https://github.com/izu0421/yytools).

### Analysis of single cell data and cell type-specific Mendelian randomisation

For the analysis of *MTHFD2L* expression in single cell RNA sequencing data, the processed sequence reads [25] were analysed using Seurat v3 [72]. This dataset profiled 10 female and 9 male donors. Previously published embeddings [25] were used to generate the UMAP. *MTHFD2L* expression was detected in 21,340 cells, which were selected in the downstream analysis. Within the selected cells, 9173 (42.99%) belonged to AD patients and 12,167 (57.01%) cells to control patients. The levels of *MTHFD2L* were then log2-transformed. Next, we used a linear regression of the data for the single neurons to query the association between the expression levels of *MTHFD2L* and AD status, accounting for age and sex. We performed additional analyses using linear mixed models that accounted for inter-individual differences in gene expression, as well as adding more covariates such as mitochondrial gene expression levels and cell subtypes.

For the cell type-specific Mendelian randomisation analysis, we incorporated the exposure data for cell-specific expression quantitative trait loci (eQTL) [73]. We ran Mendelian randomisation analyses as described in the previous paragraph, based on previously established methodologies [68, 70]. We only considered statistically significant eQTL linked to *MTHFD2L* expression in excitatory and inhibitory neurons. We combined this exposure data to the outcome data from the largest GWAS for AD [74]. We did not perform any clumping to remove potentially correlated SNPs. We used the inverse variance weighted method to model the effect of *MTHFD2L* expression in neurons on AD risk.

### Statistical analyses

Statistical analyses were performed using R version 4.0.2 and GraphPad Prism (www.graphpad.com). The data are presented as the mean values, and the error bars indicate ± the SDs. The number of biological replicates per experimental variable (n) is indicated in either the respective figure or figure legend. No power analysis was performed to assess the minimum sample size needed for hypothesis testing. The selection of sample size in individual experiments was determined based on the information gathered from assays conducted previously by our group. No sample was excluded from the analysis unless otherwise stated. Blinding was not performed. Shapiro-Wilk and F-tests assessed normal distribution and variance similarity. When these assumptions have not been met, this is stated in the figure legend. All the data collected was filtered for outliers using the ROUT method with FDR set to 1%. Significance is indicated as * for *P* < 0.05, ** for *P* < 0.01, *** for *P* < 0.001, **** for *P* < 0.0001 and NS for *P* ≥ 0.05.

### Digital image processing

Fluorescence images were acquired as uncompressed bitmapped digital data and processed using Adobe Photoshop with established scientific imaging workflows. To visualise pixel intensity, confocal images acquired with identical settings were processed using a five-tone heatmap (f.64 Academy) in Adobe Photoshop.

### Animal husbandry

The animal husbandry and experimental procedures were performed in full compliance with the United Kingdom Animal (Scientific Procedures) Act 1986. The procedures were approved by the University of Cambridge Animal Welfare and Ethical Review Body. The animals were housed in groups of up to 4 animals and kept on a 12-hour dark-light cycle with free access to standard chow and water.

## Supplementary information

Supplementary Table 1

Supplementary table legend

## Data Availability

All data for Figs. 1–8 are available in GitHub (https://m1gus.github.io/AD-FA/). The proteomics data have been deposited at the ProteomeXchange Consortium with dataset identifier PXD040263 Access to the UK Biobank data can be applied for via the UK Biobank website (www.ukbiobank.ac.uk). All other data are available upon reasonable request.

## Code availability

All code is available in our GitHub repository (https://m1gus.github.io/AD-FA/).
