# Supplementary material for: Enhancing mitochondrial one-carbon metabolism is neuroprotective in Alzheimer’s disease models
Source: Cell Death Dis. 2024 Nov 24;15(11):856. doi: 10.1038/s41419-024-07179-3 (PMC11586400; doi:10.1038/s41419-024-07179-3)
Supplement: Supplementary file 2 — Supplementary table legend [file 41419_2024_7179_MOESM2_ESM.docx]

# Supplemental Data

## Supplementary Table 1. Full list of proteins detected in Aβ-Arc-expressing flies and controls.

The individual quantitation levels are shown as normalised log-transformed (base 2) abundance values from the mass spectrometer. The statistical tests used were linear models with variances moderated by the empirical Bayes method. The Adj.P.Val corresponds to a P value corrected using the Benjamini Hochberg method. This table is related to Figure 1
